# ‘If You Don't Fight for Yourself, No One Else Will’: An In‐Depth Interpretative Phenomenological Analysis of Rare Disorder Care Advocacy in Aotearoa New Zealand

**DOI:** 10.1111/hex.70668

**Published:** 2026-04-19

**Authors:** Lucy Bennett, Tara N. Officer

**Affiliations:** ^1^ Te Puna Hauora – School of Health Victoria University of Wellington Wellington New Zealand

**Keywords:** Aotearoa New Zealand, health services research, healthcare networks, interpretative phenomenological analysis, rare diseases, rare disorders, relational mapping interviews

## Abstract

**Introduction:**

Rare disorders are not rare, collectively affecting around 300 million people worldwide. Rare disorders vary in aetiology, symptomology, and treatment, yet people living with them experience common challenges relating to diagnosis, information access, healthcare access, and support. This research explores the lived experiences of navigating networks of care for people living with rare disorders and their carers. Prior research in this area, particularly in Aotearoa New Zealand, is scant, meaning that it is difficult to clearly understand experiences of navigating rare disorder care and how they might be improved.

**Methods:**

Eleven people living with rare disorders and four carers were recruited to participate in interviews through the Rare Disorders New Zealand Facebook page. Relational mapping interviews were used, with participants speaking about their networks of care, while also drawing maps to represent them. The analysis of these data was informed by interpretative phenomenological analysis.

**Results:**

Participants described their roles in advocating for rare disorder care. People living with rare disorders often saw themselves as isolated and experiencing the brunt of their condition, while carers depicted the isolation as conjoint. Participants acknowledged that advocacy was a central element of their experiences but described it as a reluctant fight or a responsibility they had to take on, while also identifying the unique challenges of advocacy in the context of Aotearoa New Zealand.

**Conclusion:**

This research has implications for rare disorder policy; strategies are suggested that could be implemented to improve rare disorder care, including increasing the information and support available to people living with rare disorders and their carers in Aotearoa New Zealand, to validate and strengthen their roles as advocates. It also lays a foundation for future research into rare disorder care experiences, highlighting the value of multi‐method data collection approaches and the need for more research around areas of intersectionality.

**Patient or Public Contribution:**

Rare Disorders New Zealand, the national representative body of people living with rare disorders and their families, supported this research, including its promotion through their social media channels. Participants could review and amend their data, and a Māori advisor was available to guide data analysis from Māori participants.

**Abbreviations**
GET: group experiential themes
IPA: interpretative phenomenological analysis
PLWRD: people living with rare disorders

## 1 Introduction

Around 300 million people worldwide live with a rare disorder [1]. Medically, rare disorders vary widely in aetiology, symptoms, and treatments, but those affected often face similar challenges in diagnosis, information access, healthcare, and support [2]. Most genetic disorders are rare, but rare disorders also include rare cancers, infectious diseases, immune‐related conditions, poisonings, idiopathic diseases, and undetermined conditions [3]. Rare disorders are often chronic, progressive, and life‐threatening [3]. Due to limited information, people living with rare disorders (PLWRD) struggle to access treatments, diagnoses, therapies, and appropriate health and social care. Regardless of medical differences, these populations face many common barriers [2].

PLWRD access healthcare more frequently than the general population, while also facing additional barriers such as travel distance, costs, lack of services, referrals, and long wait times [4, 5, 6]. When accessing services, they report dissatisfaction with healthcare professionals' knowledge and support, and are negatively impacted by poor service coordination [7, 8, 9]. PLWRD and carers also report that information about their disorders and available support can be difficult to find [8, 9], and the information they do have access to is insufficient [4, 10].

PLWRD and carers also report inadequate psychological and social support, despite having high needs [4, 11]. Literature suggests that PLWRD and their carers feel emotions such as disappointment [12], helplessness [13, 14, 15], powerlessness [16, 17] and frustration [18, 19] when navigating rare disorder care. Anxiety, depression and stress are also common experiences for PLWRD and carers, as reported in several studies [11, 12, 15, 20, 21, 22, 23, 24, 25, 26].

### 1.1 The Aotearoa New Zealand Rare Disorder Landscape

People living with rare disorders and carers in Aotearoa New Zealand (hereafter Aotearoa) encounter significant challenges [27], but there is limited information available on how these challenges are experienced. A 2023 survey [27] revealed that PLWRD in Aotearoa use healthcare services extensively – 85% saw a specialist and 91% visited a general practitioner in the 180 days before completing the survey. Many respondents found support services inadequate, with 51% saying services were poorly prepared to help them, and 64% noting that services had limited knowledge about their disorder. Additionally, 79% felt uninformed about their rights, 73% about social services, and 66% about financial entitlements. Isolation was common among respondents, with 69% feeling distanced from family and friends. Mental health concerns included 36% of people often feeling unhappy or depressed (compared to 11% in the general population) and 34% often feeling unable to overcome problems (compared to 8% in the general population). The results from this survey are comparable with many international surveys, including a European survey with similar questions [28]. While not worse, they are also not better than the findings from other countries, highlighting a need to develop awareness, understanding and evidence around the experiences of PLWRD and carers in Aotearoa.

In 2024, the Ministry of Health published the *Aotearoa New Zealand Rare Disorders Strategy*, which sets the direction for the health system in Aotearoa to support PLWRD and their whānau (family) [29]. The strategy cites the ongoing importance of understanding the lived experiences of PLWRD and their carers as the strategy moves into an implementation phase. It also sits within a broader global context of recognition for rare disorders, with a 2021 United Nations Resolution calling for improved healthcare for PLWRD [30].

National strategies for rare disorders vary worldwide, offering different levels of recognition, support, and healthcare [31]. The present research aims to illuminate what it is like to live with or care for someone with a rare disorder in Aotearoa. International literature does not fully capture the experience of living with rare disorders in Aotearoa, a country with a small population, fragmented health system [32], unequal access to specialist services [33, 34] and recently developed Rare Disorders Strategy [29]. Additionally, existing survey data does not allow for in‐depth, qualitative analysis of the experiences of PLWRD and carers in Aotearoa. To address these gaps, this research answers the following questions:

1. What are the lived experiences of navigating networks of care for people living with rare disorders, and/or their carers, in Aotearoa?
2. How do people living with rare disorders in Aotearoa, and/or their carers, perceive strengths, weaknesses, and opportunities for change within these networks?

## 2 Materials and Methods

Interpretative Phenomenological Analysis (IPA) was chosen as the methodology for this research. IPA is underpinned by phenomenological, hermeneutic and idiographic theories, which can help to facilitate in‐depth explorations of complex topics [35]. Phenomenological research focuses on lived experiences [35, 36, 37], prioritising, in this case, the perspectives and understandings of PLWRD and carers themselves. Hermeneutics allows for an exploration of how these experiences are interpreted, enabling researchers to explore the depth of participants' lived experiences as they understand them, particularly useful in a consistently overlooked and underserved population [38, 39]. Finally, an idiographic lens allows researchers to focus on a specific group and their experiences, rather than generating more general ideas about people's experiences [35, 39]. IPA can be useful when exploring complex and emotionally‐laden topics [38], such as experiences of navigating rare disorder care. IPA studies that have been undertaken with PLWRD have illuminated the psychological, social and contextual factors inherent to this experience (for example, [40, 41, 42]). This insight can help caregivers, health and social support professionals, and policymakers understand how they can provide meaningful support to PLWRD [40, 41]. Ultimately, IPA researchers explore lived experience in depth, focussing on how people make sense of experiences [43, 44].

## 3 Methods

Participants were invited to contact the lead author (LB) through an invitation to an interview posted on the Facebook page of Rare Disorders New Zealand, the only national representative body of PLWRD and their families. The invitation was open to adults (over 18 years old) who lived in Aotearoa and lived with or cared for someone with a formal rare disorder diagnosis. The inclusion of PLWRD and carers was due to the project's focus on navigating rare disorder care, rather than living with a rare disorder. As 50% of rare disorders affect children [45], navigating rare disorder care is managed by caregivers for many in the rare disorders community. Therefore, both groups were included so as not to exclude this considerable proportion of the population.

Forty‐five people expressed interest by responding to the initial invitation. Following the lead author reaching out to those who expressed interest, sharing information sheets and consent forms, and discussing availability for interviews, 15 people confirmed that they would participate. Recruitment was stopped at this point to maintain project manageability; however, interested participants were advised that they may be approached later if there was not sufficient in‐depth experiential richness and meaningful pattern identification across data from participants already scheduled for interview. Although 15 participants are a larger than usual sample size for IPA [35], all participants who confirmed they wished to participate were included. This was in line with the project's aim of amplifying an often‐unheard voice, and the authors chose not to arbitrarily exclude participants who wanted to share their stories.

Each of the 15 participants chose their own pseudonym and provided key demographic information through a standardised form either before or during the interview (Table 1). Participants were informed that pseudonyms would be used to protect their privacy, although some expressed a preference to be identified by their real names. In these instances, we outlined the associated risks and confirmed the use of pseudonyms as a consistent protective measure across the dataset. Although most participants disclosed their rare disorder during the interview, this information was not included to protect confidentiality. As Aotearoa has a small population of 5.2 million people, few people may have each rare disorder, making them potentially identifiable if the disorders were to be specified.

**Table 1:** Participant demographics.

Pseudonym	PLWRD or carer	Age range (Years)	Ethnicity	Gender
Alison	PLWRD	45–54	British	Female
Anna	Carer	45–54	New Zealand European	Female
Chris	PLWRD	55–64	Australian	Male
Dylan	Carer	35–44	New Zealand European, German	Female
Gill	PLWRD	55–64	New Zealand European	Female
Hilary	PLWRD	65+	New Zealand European	Female
J	PLWRD	55–64	Not disclosed	Female
John	Carer	65+	New Zealand European	Male
M Weasel	PLWRD	25–34	New Zealand European	Female
Mrs Bucket	PLWRD	45–54	New Zealand European	Female
Rachel	PLWRD	35–44	New Zealand European	Female
Rog	Carer	55–64	New Zealand European	Male
Rose	PLWRD	55–64	New Zealand European	Female
Stella	PLWRD	35–44	Latin American	Female
Steve	PLWRD	45–54	New Zealand European	Male

All participants had accessed rare disorder care through Aotearoa's healthcare system. The four participants who were carers all cared for their own children, and these children's ages ranged from very young to adult. Additionally, Steve brought a support person, Shelley, to his interview, who consented to her input being included.

Participants provided written informed consent before taking part in this research. LB conducted all interviews; these lasted between 33 and 80 min. Nine were Zoom (Zoom Communications Inc.) based; three were in‐person, and three were over telephone, reflecting participants' preferences. A field log was kept throughout data collection and analysis to evaluate the ethical, methodological, and reflexive practices used.

Data was collected using Relational Mapping Interviews [46], which combine semi‐structured interviews with a drawing task where participants map their healthcare networks. This method is structured around four touchpoints, wherein participants map themselves, map important others, stand back, and consider change [46]. The draw‐talk‐draw‐talk format encourages participants to visualise and then elaborate on their healthcare networks, providing complex relational insight [47].

For in‐person interviews, paper and drawing materials were provided. For online interviews, participants created maps beforehand or used the Zoom Whiteboard feature during the interview. Participants interviewed over telephone created their maps before the interview and sent copies to LB. Materials for in‐person interviews matched Zoom Whiteboard options to ensure consistency. Full instructions were provided to prepare participants and ease any concerns, following Boden & Larkin's [46] suggestions. All methods were pilot tested before the interview.

### 3.1 Analysis

There is no definitive way to conduct IPA research, but there are flexible guidelines for analysis [35]. These guidelines informed development of analytical steps. Field notes were made immediately prior to and following each interview to enhance reflexivity. Each interview was then transcribed using Otter AI software (Otter.ai, Version 2.18.3), checked for accuracy by the lead author, and reviewed to identify interesting aspects. Participants also had the opportunity to review transcripts. Although unused due to a lack of Māori participants, a Māori advisor with experience working in the rare disorders space was also available to guide analysis of Māori participants' interviews. Preliminary lists of Personal Experiential Themes were made for each transcript before being combined into Group Experiential Themes (GET). These GET were loaded into NVivo (Lumivero, Version 12.1.1) as codes. Each transcript was coded into the GET framework, with notes reflecting developments in how GET were being understood. The most relevant GET were then identified, combined (in some cases), and organised into themes. To ensure analysis was informed by participants' experiences, previous stages, particularly initial field notes, were referred to during and after analysis. The research team discussed and refined preliminary findings iteratively, meaning subsequent interviews were informed by preliminary analyses.

Relational maps were analysed in their own right. Questions drawn from a framework initially described in Boden & Eatough [48] and further developed in Boden et al. [47], were used, including:

1. What are the overall characteristics of the map?
2. How is the participant represented?
3. How are other people represented?
4. How are relationships represented?
5. What is the overall tone and impression of the image?

Each map was analysed in relation to these questions using a spreadsheet, along with more specific sub‐questions regarding, for example, colour, symmetry, focus, symbolism, materials, and style (22 questions in total). GET were developed for the maps and compared with GET developed from the textual data. The dialogue between visual and textual data was used to refine results.

## 4 Results

**Figure 1:**
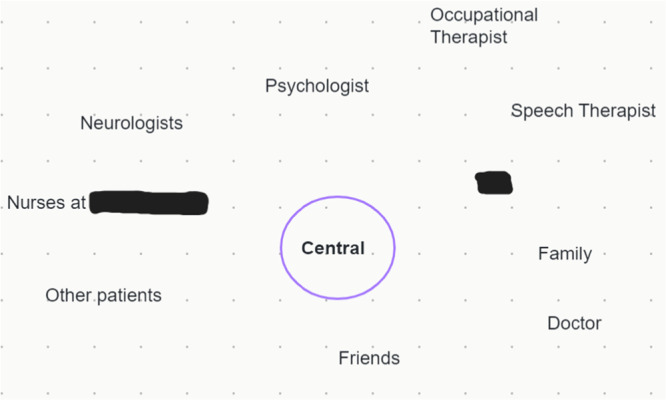
Rose's relational map.

The positioning of PLWRD was linked to their struggles to reclaim the right to be at the core of their care. For instance, Rose emphasised her centrality by representing herself using the word ‘*Central*’ within a circle on her map, highlighting her need to self‐advocate continuously (Figure 1).

**Figure 2:**
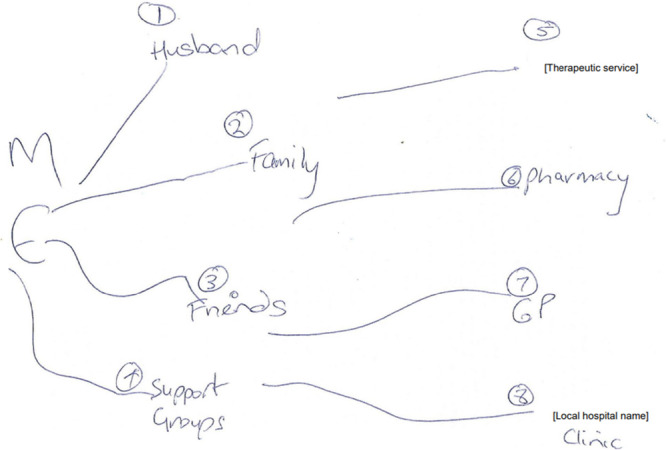
J's relational map.

Similarly, J used a tree diagram with ‘*ME*’ as the root, signifying that all support stemmed from her efforts to keep herself as the ‘*focus*’ of her rare disorder care (Figure 2). PLWRD were often alone at the centre of their maps, evoking a sense of isolation and sole responsibility.

While PLWRD often saw themselves as isolated at the centre of their rare disorder care, experiencing the brunt of their condition, carers tended to depict it as a collaborative effort. They discussed the collective impact and shared responsibility defining their rare disorder experience. Rog noted that he and his wife played equally crucial roles in managing their son's care, emphasising their ‘*hands‐on approach for most of [their child's] life*’. Shelly, the partner of Steve, who had a rare disorder, explained how their family was collectively impacted by Steve's condition, with everyone affected in various ways and constantly involved in his care. Similarly, Dylan described how their family had always navigated their child's rare disorder journey ‘as a family’.

By placing themselves centrally, participants demonstrated their own importance in the context of rare disorder care. The results discussed in this paper are drawn from participants' mapping of themselves in their interviews, and are structured around the concept of self‐advocacy. In this research, self‐advocacy encompasses pushing for appropriate and timely healthcare, obtaining accurate and sufficient information, and seeking respect within healthcare interactions. Three themes were identified that highlight how PLWRD and carers in Aotearoa experience advocacy: a reluctant fight; a responsibility; and challenges in Aotearoa. The first two themes apply to PLWRD and carers, respectively, while the third theme demonstrates their shared experiences.

### 4.1 Advocacy As a Reluctant Fight

For PLWRD, self‐advocacy was often described as an unavoidable fight. This was captured by Rachel's view that *‘if you don't fight for yourself, no one else will’*. Other participants echoed this sentiment, including Gill (*‘I have to take control of my own health’*) and Rose (*‘I've had to advocate the whole way for myself’*). While being a self‐advocate was described as necessary for PLWRD, it was also described as a role that they had no choice but to take on.

Participants, such as Mrs Bucket, felt discomfort in advocating for themselves. She explained that she was *‘not so good at fighting for myself, but I have for other people… I could stand up for somebody else. But I don't find that so easy for myself’*. However, she still acknowledged the necessity of fighting for her care. Conversely, Hilary chose to *‘minimise things’*, finding it *‘easier’* than pushing for help. Although she found it difficult that *‘nobody is knowledgeable’*, she expressed that she was *‘not expecting miracles’*. These participants' experiences exemplify the burden of self‐advocacy, which, in Hilary's case, was simply too much to take on.

PLWRD were also responsible for independently finding information about their disorders to be able to advocate for themselves. Hilary described how she navigated *‘endless information’* through patient support groups:

There's a whole network of people who communicate with each other, ask questions… I talk to some of these people, sometimes they do Zoom sessions… and they do a weekly sort of, club house they call it, where people can just say how they're getting on. Apart from that, it is an awful lot of YouTube videos, lectures from all sorts of specialists.

Rachel emphasised the diverse expertise found within these groups, describing them as ‘*so full of information*’, including ‘*recommendations for medicine, information on medicine and side effects, and just tips, like so many tips*’. The extent of information sharing in these groups suggests that individuals rely on peer‐to‐peer networks, rather than healthcare professionals, for accessing information. PLWRD are responsible for educating themselves to self‐advocate, adding another dimension to the role.

However, information gathering was daunting for some, particularly when support groups could not give necessary advice. J illustrated the relentless nature of this search:

I go hunting if I think I really need such and such. And I'm going to find out, I'm going to find it. And so that's what I do, and if I can't find it, then I'll find somebody that can look into it a bit more for me, or then I have to figure out something different.

Mrs Bucket's post‐diagnosis experience underscores the emotional toll and alienation felt when left to navigate rare disorders alone:

When I first got diagnosed with the specialist, well, the next time we went to the doctor, I said, "could I have a bit more information on the condition?" And she told me to go online, because you'll find more information on there than what I can give you. And I felt so lost.

Ultimately, the struggle of self‐advocacy for PLWRD highlights the burden placed on them to navigate and manage their own health in the absence of adequate support from the wider healthcare system.

### 4.2 Advocacy As Responsibility

Carers also described advocacy as a key responsibility of navigating rare disorder care, although they tended to discuss it more as a duty or profession than a burden. This may be related to participants having professional experience in advocacy roles, or the fact that caring for a child with a rare disorder often replaced some or all of the parents' paid employment time. This was encapsulated by Rog, who described his experience of giving up work to look after his son as a career transition from the financial industry to healthcare advocacy. He shared how he and his wife became knowledgeable about healthcare system intricacies, eventually leading him to take on a formal consumer advocate role.

Other parents also took pragmatic approaches to advocacy, with Anna describing her role in her daughter's care as managerial – *‘I'm in charge of it’*. She shared how she had taken on an array of new responsibilities, including *‘advocate, helper, everything. Diagnoser… Supporter, all those things. Nagger’*. John also self‐identified as an advocate for his son, attributing this to his professional‐level knowledge of the rare disorder – surpassing what healthcare professionals provided. For these parents, as for PLWRD, advocacy was vital in securing necessary care. However, it was not primarily seen as a burden, but as an extension of their parental, and often professional, responsibilities.

While finding information was a major responsibility of carers as well, they also emphasised their responsibility managing information in relation to their child's rare disorder. For example, John and Rog discussed the considerable effort they put into preparing and maintaining their children's medical records. John explained how he condensed details of his son's disorder into card‐sized summaries:

We've trained a lot, a lot of people…at one stage we had a little, we got it down to a, probably about ¼ of a page, you could almost print it on a card… if we had to take them to the emergency department or anything like that, it was sort of like, here's six bullet points about what their condition is because some, we'd look at the doctors you know, you'd almost see them go "oh my God. What's this?"

Rog echoed the need for preparedness, keeping a medical file *‘about three inches thick’* alongside digital records. He worried about the continuity of care when his GP retired: *‘do you then have to repeat everything? How well, how good are the notes that are being kept?’* The uncertainty around who updates records made parents feel like the only reliable keepers of their child's medical history. Rog emphasised the importance of maintaining these documents, ensuring that if something happened to him and his wife, records would be available for whoever took over their child's care.

However, for carers who had not been immersed in the healthcare system for as long as John and Rog, finding information about rare disorders still felt impossible. Dylan described the experience as having to *‘pull information here and there out of this, like, big box of like, black question marks… bleak black question marks’*. While Dylan also managed complex webs of healthcare professionals for her child's care, and saw herself as the expert among them, this was a position she struggled to maintain. This suggests that, for carers, being expert advocates may be something that develops out of necessity.

For carers, advocacy involves pragmatic management of their children's care and medical information and was often described as a professional‐level responsibility. While it can still be daunting, the role of an advocate is an essential part of ensuring the wellbeing of those for whom they care.

### 4.3 Challenges of Advocacy in Aotearoa

Perceptions of cultural attitudes towards healthcare advocacy in Aotearoa highlight the shared struggles of PLWRD and carers in navigating rare disorder care. For example, Rog, reflecting on his work as a consumer advocate, noted:

New Zealand's a lot more laid back. We don't, we're not as bullish… If you get a group of Aussies [Australians] in a room and a group of Kiwis [New Zealanders] in the room, the Aussies will be a bit more forthcoming and outspoken… New Zealand's a lot more laid back, and we don't sort of ask the hard questions. And we don't complain as much. And I think because of that, the status quo is what continues for a lot of health scenarios.

This culturally characteristic ‘laid‐back’ disposition, together with awareness of pressures and long wait times in Aotearoa's publicly funded health system, may contribute to participants' discomfort when required to advocate for their own care. Participants often described self‐advocacy as an exhausting necessity rather than an empowered choice. Hilary, for instance, admitted that she avoided advocating for herself simply because it was *‘easier’*. Taking on the battle for adequate care was not automatic, but rather something with which people had to engage consciously.

Shelley's experience with her partner's medical care exemplifies the discomfort associated with assertiveness in healthcare settings. She pushed hard for Steve's treatment, encountering resistance from medical professionals who did not expect her to be so *‘bolshie’*. While she did not feel apologetic for her assertiveness, she acknowledged that it brought out an unfamiliar side of her. Similarly, John, despite his confidence in advocating for others, admitted to feeling *‘crushed’* when facing opposition as he fought for his son's care. His experience illustrates how advocating for care can be emotionally draining, even for those practised in advocacy.

Across participants, self‐advocacy was rarely described as intuitive or easy. Participants described their attitudes in standing up for themselves as *‘bolshie’* (Shelley, Gill), *‘pushy’* (Rog, John) or having to *‘fight’* (Mrs Bucket, Dylan, Rachel, Rose, MW). Whether rooted in cultural expectations or the inherent discomfort of challenging medical authority, New Zealanders navigating rare disorders find themselves forced into advocacy, often reluctantly.

Similarly, PLWRD and carers in Aotearoa described being united in their experience by the scarcity of local information and support networks. Hilary, for instance, sought help from an Australian support group due to the inactivity of her Aotearoa‐based one, but found there was *‘too much information’* compared to *‘not enough information’* in the Aotearoa‐based group. Rachel had a similar experience, noting that local support groups lacked engagement, and that *‘you might get one or two people replying’* to most of her questions. To overcome these barriers, participants turned to international groups or broader support groups for other disorders where people might be experiencing similar challenges.

Alison also found online support groups useful, but recognised their limitations. Many online discussions focused on healthcare systems in other countries, particularly the United Kingdom, making much of the advice irrelevant to Aotearoa health service delivery. Though the medical realities of rare disorders remain constant worldwide, treatment and support access varied, creating added challenges for those in Aotearoa.

Advocating for rare disorder care in Aotearoa meant that PLWRD and carers reached further for reliable information and pushed harder than is culturally expected for sufficient care. Their positioning in Aotearoa unites both PLWRD and carers in shared struggles, reinforcing the need for resilience in a system without accommodation.

## 5 Discussion

Despite growing recognition of rare disorders worldwide, Aotearoa's healthcare system has failed to keep up with the needs of PLWRD and their carers [49]. This research illuminated experiences of PLWRD and carers in Aotearoa and is one of the first qualitative explorations of how they navigate healthcare in this context. Examining experiences collectively, rather than through individual diagnoses, highlights shared challenges faced by the rare disorders' community. Further, discussing the experiences of PLWRD alongside the experiences of carers allows for an exploration of the nuances of the advocacy role between these two groups. Prioritising the voices of PLWRD and carers is essential in naming barriers to care and ensuring realities are accurately understood.

PLWRD and their carers often find themselves positioned at the centre of their healthcare journeys as self‐advocates and coordinators of their own care. Being at the core of their care allows PLWRD and carers to assert expertise, advocate for themselves or those they care for, and actively manage medical care. While these roles can foster strength and determination, isolation participants experienced in the central position highlights systemic care gaps. While PLWRD and carers' ability to self‐educate and push for proper treatment enhances the support they receive, it places sole responsibility for finding appropriate support on their shoulders. The healthcare system's reliance on their initiative underscores fundamental rare disorder care weaknesses and an imbalance in the responsibility for rare disorder care.

The necessity for self‐advocacy from PLWRD and carers is reflected within rare disorder literature undertaken in other countries [50, 51, 52]. While this is sometimes framed as a positive experience, helping people feel empowered [53, 54], proud [55], and heroic [13], these experiences are often undercut by the ongoing necessity, rather than choice, of self‐advocacy [51, 52]. This research expands on these findings by examining both PLWRD and carers together, revealing the emotional and practical complexities of advocacy in rare disorder care. PLWRD in the present research were more likely to describe self‐advocacy as a burden, while carers framed it primarily as a responsibility. However, they had many shared experiences, particularly when it came to the discomfort associated with self‐advocacy. Regardless of whether they were a PLWRD or carer, had previous advocacy experience, or had been navigating rare disorder care for a short or long period, a prevailing point among participants was that self‐advocacy should not be such a necessary aspect of accessing appropriate healthcare. In this sense, the themes of burden and responsibility are two sides of the same coin, both reflecting the inequitable nature of rare disorder care.

A unique finding of this research was participants' descriptions of acting in ways that went beyond what they saw as expected behaviour in Aotearoa, to stand up for themselves and their own expertise with healthcare professionals. Parents adopted a professional demeanour with regard to managing their children's care, suggesting it was a role that required more from them than their normal parental duties, while other participants chose to take easier roads and not advocate for themselves. This expands on findings in existing literature on the anxiety that PLWRD and carers feel about the responsibility of finding their own information, the lack of support that accompanies it, and the sense of discomfort associated with asserting their own knowledge to healthcare professionals [19, 53]. The present research situates experiences of self‐advocacy within perceptions of acceptable behaviour in Aotearoa, illuminating contributing factors to PLWRD and carers' discomfort in this context. Healthcare professionals should be aware of the cultural discomfort felt by PLWRD and carers in advocating for themselves, to provide them with appropriate care – including the care for which they may not directly ask.

Participants in this research emphasised their struggle to find relevant information on their rare disorders in Aotearoa. Existing literature highlights how individuals bear the burden of educating themselves and healthcare professionals involved in their care [19, 50, 56]. PLWRD and carers describe worrying about the quality of the information they find, and are aware of the danger of misinformation [12, 20, 57]. When information is available, it may contain language and material inaccessible to non‐healthcare professionals, or be subjective and fragmented [20, 23]. The findings of the present research suggest that the issues described in the wider literature may be exacerbated in Aotearoa, due to small population size and geographical isolation.

While PLWRD found empowerment in advocacy, they also found burden. A more interconnected support system, along with increased awareness among healthcare professionals, could provide greater assurance that PLWRD are not solely responsible for advocating for themselves and educating those around them. This would also help alleviate medical mistrust fostered by the lack of awareness surrounding rare disorders. Munro et al. [51] describe participants' experiences of treatment delays due to doctors' unwillingness to listen to PLWRD, resulting in PLWRD becoming disabled and narrowly avoiding fatal outcomes. Bush et al. [54] and Uhlenbusch et al. [57] similarly describe how participants felt medically uncared for in terms of their rare disorder and were unable to trust healthcare professionals due to limited disorder‐specific understanding. With better resources and improved awareness about rare disorders in Aotearoa, PLWRD and carers may feel less isolated, making healthcare experiences more manageable and equitable.

### 5.1 Recommendations

IPA focuses on the lived experience of participants, an understanding of which can contribute to service development and evaluation [58], but the methodology does not explicitly draw out recommendations from participants. The following recommendations, therefore, have been produced by the authors based on the experiences participants shared when they were asked to ‘consider change’ as part of this research. There is a wealth of expertise among PLWRD and their carers that should be formally recognised in policy. Establishing rare disorder advisory groups within health, disability, social welfare, and education sectors could integrate insights into decision‐making processes. Research into PLWRD engagement in policy development has shown that involvement can amplify an otherwise ‘weak’ voice, and reframe commonly experienced issues as systemic, rather than individual, issues [59]. Rare Voices Australia notes the importance of credibility for effective political advocacy [60]; a sustainable, well‐funded platform through which PLWRD and carers advocate for themselves would affirm their skills and ensure rare voices influence policy decisions. Formal recognition may further support recognition of PLWRD and carer rare disorders clinical practice expertise, without the need for patients to feel required to fight for care without discrimination, something that should be a fundamental right [61, 62].

Recognising the advocacy fight described by our participants, increasing funding for rare disorder support and advocacy organisations would also strengthen the resources available to support PLWRD. Removing reliance on contestable funding rounds [63] would enhance the ability for organisations to provide guidance, raise public awareness, and ensure consistent support. Countries with strong rare disorder patient advocacy organisations have a strong influence on rare disorder awareness, policy and health system response [64]. Activities they undertake include implementing programmes and support, and calling for legislation to better support their needs [64]. Rare Disorders New Zealand has called for their inclusion as a key partner in implementing the Aotearoa New Zealand Rare Disorders Strategy [27], but without greater recognition and support of the role of advocacy organisations, they are limited in what they can provide.

Lack of awareness and understanding of rare disorder care needs poses significant challenges for PLWRD and their carers. Time and effort are spent searching for, interpreting, and managing medical information, which slows down diagnosis and treatment. A dedicated, centralised hub for information on rare disorders navigation could alleviate these burdens and improve healthcare accessibility in Aotearoa. This could be modelled off, and perhaps linked to, examples such as Orphanet, an international resource offering extensive inventory of rare disorders, expert directories, patient organisations, and emergency medical guidelines [65]. More localised examples include Rare Voices Australia's RARE Portal, funded by the Australian Government, which provides information and resources on rare disorders customised for an Australian context [66]. Alongside this, Rare Voices Australia runs a RARE Helpline available to answer questions about rare disorders and navigating care [67]. The National Organization for Rare Diseases, based in the United States, hosts a Rare Disease Database on its website [68]. This provides information and resources on specific rare disorders, including reports created by the Organization and compiled from other sources. A similar Aotearoa‐specific platform could be developed to provide local information, including directories of specialists, rare disorder support groups, clinical research, and healthcare navigation tools. Ensuring accessibility for PLWRD, carers, advocates, and healthcare professionals would allow for greater collaboration and efficient knowledge‐sharing. Acknowledging challenges with rare disorders expertise in a small country such as Aotearoa, such a platform could serve two important purposes, it could (1): limit the need for PLWRD to recreate navigational journeys others have been on, and (2) offer an Aotearoa‐specific platform, acknowledging that joining or pooling resources with other health systems would be of limited value if the population could not access or relate to the opportunities described in these resources.

### 5.2 Limitations and Future Research

Given the genetic aspect of many rare disorders, diverse perspectives are crucial. Despite efforts to capture diverse participant bases, this research did not include Indigenous Māori, or Pasifika or Asian participants (two growing populations in Aotearoa [69]). Aotearoa's healthcare system often fails to meet Māori needs [70, 71], meaning this population is likely to face compounding healthcare access inequities. To honour *Te Tiriti o Waitangi* (the founding document of Aotearoa) and address compounded inequities, future research should consider adopting a kaupapa Māori research methodology approach to prioritise Māori perspectives and promote Māori wellbeing and knowledge. Kaupapa Māori methodology requires Māori leadership and deep grounding in mātauranga Māori (Māori knowledge) [72]. Similarly, using Pacific methodologies requires extensive relationship building within communities and a grounding in Pacific worldviews [73]. The research team recognises that such methodologies, grounded in specific leadership and community relationships, present opportunities to capture important cultural nuances beyond what we have identified in the general Aotearoa population. Applied internationally, our research suggests the need for further research around areas of intersectionality between demographic and social needs, and rare disorders care.

Participant engagement in the mapping exercise varied, and the approach could be strengthened in future research. For participants who were engaged in the mapping exercise, the maps stimulated conversation, elicited unexpected reflections and produced valuable visual data that added context and nuance to the interview data. However, other participants appeared to find the activity distracting and preferred instead to focus on conversations with the lead author. The more engaged participants tended to be those using physical drawing materials instead of software, who were more likely to switch colours, materials, and shapes; use space more fully and draw more expressively. For future online interviews, asking participants to prepare maps in advance would allow them to reflect on their representations during interviews, rather than struggling with unfamiliar software while discussing experiences. Nonetheless, we encourage future researchers to adopt multi‐method approaches to qualitative data collection to support participants in providing their stories of advocacy.

International studies have explored how healthcare for PLWRD and carers overlaps with non‐medical services, including social care, disability support, and education [18, 74]. Participants in the present research discussed these overlaps, but it was outside scope to explore the impact of non‐healthcare services. Future studies should, therefore, explore overlaps between other systems, such as education and social welfare, to ensure holistic support for PLWRD and their carers.

Finally, this research focused on participants' subjective perceptions of experiences, and the findings should be understood as such. In line with the aims of IPA methodology, we sought to explore how PLWRD and carers experienced care and made sense of those experiences, rather than produce generalisable results. We view this as a feature, rather than a limitation, of IPA research, but also recognise that IPA research can sit alongside other methods and methodologies to create layers of exploration and explanation [35]. We recommend future research that contributes to this dialogue, particularly exploring healthcare system mechanisms that shape experiences described in this research.

## 6 Conclusion

The experiences described by PLWRD and carers in this research underscore the need for systemic healthcare system transformation. To our knowledge, our work is the first of its kind in Aotearoa to describe these experiences. Findings reveal the emotional toll and discomfort PLWRD and carers feel when self‐advocacy becomes necessary to access needed care. Building sustainable advisory groups, investing in rare disorder advocacy organisations, and developing accessible, localised information platforms are important to addressing challenges identified through this research. Enhancing equitable care means removing burdens of self‐advocacy from individuals and promoting a coordinated, responsive health system that recognises rare disorders as deserving of informed and inclusive care.

## Author Contributions

**Lucy Bennett:** conceptualization, methodology, validation, formal analysis, investigation, data curation, writing – original draft, writing – review and editing, project administration. **Tara N. Officer:** conceptualization, methodology, validation, writing – review and editing, supervision, funding acquisition.

## Ethics Statement

In accordance with the National Ethical Standards for Health and Disability Research, the Te Herenga Waka – Victoria University of Wellington Human Ethics Committee granted this project ethical approval on 2 May 2023 (#0000030868). Written informed consent was obtained from all participants in this research.

## Conflicts of Interest

LB is a past employee of Rare Disorders New Zealand (RDNZ) she is also an employee of the New Zealand Ministry of Health, but has not contributed to this project as a Ministry employee. TNO is a member of the RDNZ Rare Disorders Research Network Leadership Group.

## Data Availability

The datasets generated and analysed during the current study are not publicly available due to maintaining participant confidentiality and anonymity.
